# Sedentary Activities and Food Intake among Children and Adolescents in the Zhejiang Province of China: A Cross-Sectional Study

**DOI:** 10.3390/nu15173745

**Published:** 2023-08-26

**Authors:** Yan Zou, Lichun Huang, Mengjie He, Dong Zhao, Danting Su, Ronghua Zhang

**Affiliations:** Zhejiang Provincial Center for Disease Control and Prevention, Hangzhou 310051, China; zouyan0573@163.com (Y.Z.);

**Keywords:** sedentary behavior, food, intake, children, adolescents

## Abstract

Background: Sedentary behavior may affect the types of food consumed in children and adolescents’ daily diets. Previous published studies are limited to local surveys. This study aimed to explore the relationship between sedentary behavior and food intake among children and adolescents. Methods: A stratified sampling technique was employed in the present cross-sectional study. Demographic characteristics, sedentary behavior, transportation modes, and food intake were investigated. Results: We found that children and adolescents who watched movies or TV programs online or on their smartphones on weekends and who chatted online on weekends, including on QQ (an instant messaging software service) and WeChat (an instant messaging software service), increased their intake of instant noodles and fried pasta (Spearman’s rho = 0.468, 0.575, 0.465, and 0.323; *p* < 0.05). Children and adolescents who chatted online on weekends, including on QQ and WeChat, increased their intake of tofu skin (Spearman’s rho = 0.461; *p* < 0.05), and those who browsed online on weekdays increased their intake of whole-fat liquid milk (Spearman’s rho = 0.455; *p* < 0.05). Children and adolescents who browsed and chatted online on weekends, including on QQ and WeChat, and who played computer or smartphone games, increased their intake of fried potato chips (French fries or other fried snacks) (Spearman’s rho = 0.568, 0.270, and 0.412; *p* < 0.05). With respect to modes of transportation used to travel to and from school, children and adolescents who took buses and subways increased their intake of rice, instant noodles, sweet potatoes, soybean milk, tofu skin, processed meat products (sausage, ham sausage, or lunch meat), fish, shrimp, vegetables, nuts, and sweet cookies (buns, cakes, Dim sum, and moon cakes) (Spearman’s rho = 0.394, 0.536, 0.630, 0.408, 0.485, 0.441,0.410, 0.424, 0.444, 0.541, and 0.366; *p* < 0.05). Conclusions: Sedentary behavior affects the types of food consumed in children and adolescents’ daily diets. Children and adolescents who browsed online on weekdays increased their intake of whole-fat liquid milk, but also increased their intake of foods with high fat, high salt, and low nutrient density. Children and adolescents taking buses and subways increased their intake of low-nutrition quality products. Public awareness efforts should focus on reducing the consumption of low-nutrition quality products and nutritional education.

## 1. Introduction

Low levels of physical activity, high levels of sedentary behavior, and poor dietary habits are important contributors to several adolescent health problems, such as obesity and cardiovascular risk factors [1]. Sedentary behavior refers to activities that are carried out in a sitting, leaning, or lying position while awake, consuming much less energy than exercise and other physical activities (such as watching TV; using computers, tablets, or mobile phones; and sitting still while taking transportation). Sitting continuously for more than one hour is considered sedentary. Sedentary activity may affect the types of food consumed in children and adolescents’ daily diets. This sedentary activity and preferences for food intake may lead to health issues, such as obesity, and can affect children’s development and long-term health. A previous review showed that diet, physical activity, and sedentary behavior tend to cluster in adolescents in a complex way, resulting in both healthy and unhealthy groups [2]. Another study on health-related quality of life and lifestyle behavior clusters in school-aged children from 12 countries highlighted the importance of a healthy combination of low screen time, healthy eating patterns, and balanced daily activity behaviors [3].

Unhealthy nutritional behaviors are related to increased body weight [4], which may cause numerous health conditions such as cardiovascular disease, diabetes, and cancer [5]. Engaging in unhealthy lifestyle behaviors increases the immediate risks of physical and mental health problems, and also increases the risk of incidence rates and mortality in adulthood, imposing significant demands on both individuals and society, particularly within health and care systems [6]. Childhood and adolescence are important developmental periods for introducing early prevention and intervention measures in terms of eating and dietary intake behaviors, as these periods are critical to the development of obesity and eating behaviors [7]. Modifying various types of nutritional behaviors during the earlier years is important to increase positive health outcomes.

Nutritional behaviors may cluster differently based on socioeconomic and demographic factors. Therefore, understanding the relationship between sedentary behavior and food intake is crucial in order to develop appropriate public health strategies and interventions among children and adolescents in Zhejiang Province, China. However, the relationship between sedentary behavior and food intake among children and adolescents under current socioeconomic and demographic circumstances is understudied, leading to a knowledge gap. This study aims to explore the relationship between sedentary behavior and food intake among children and adolescents, and to provide targeted suggestions to guide children and adolescents towards a healthy lifestyle.

## 2. Methods

### 2.1. Participants

Children and adolescents from cities and residential villages in Zhejiang Province were the subjects of this study. A stratified sampling technique was employed to select two cities and two residential villages. In every sampling unit, 140 households were selected by a random sampling method according to the household registration information. The investigators contacted the participants through household surveys. Then, at least 50 children and adolescents (aged 6–17) in every sampling unit were investigated and information on their diet intake was collected. Children with health or genetic conditions were excluded.

### 2.2. Data Collection

Demographic information, food intake, and sedentary behavior information, as well as the modes of transportation taken by children and adolescents, were collected and recorded by a trained investigator with the help of the participants’ parents. Sedentary behavior information included “watch movies or TV programs online or on their smartphones”, “chatting online on weekends, including QQ and We Chat”, “ browse online”, and “playing computer or smartphone games” during the week and weekends. Modes of transportation to school included walking to school, going to school by bus or subway, and going to school by car. Food consumption was recorded according to the Food Frequency Questionnaire, and then the daily food intake was calculated using the frequencies and weights of the food item. The food categories included staple foods, beans, vegetables, fruits, dairy, meat, eggs, and snacks. We verified the data before the analysis and excluded any implausible results, such as an energy intake of <500 kcal or >5000 kcal per day.

### 2.3. Ethical Approval

Ethical approval was obtained from the Ethical Committee of Zhejiang Provincial Center for Disease Control and Prevention (approval number: 201614). All guardians of children and adolescents provided written informed consent after the research protocols were carefully explained to them. Thus, informed consent from the parents/guardians of all participants was received.

### 2.4. Statistical Analysis

The distribution of overweightness and obesity between gender was analyzed using a χ^2^ test. As food intake and sedentary behavior time were continuous variables, the relationship between the two variables was analyzed with Spearman correlation, and Spearman’s rho test was used for statistics. Linear regression was used to analyze the time taken to go to school by bus or subway and food intake, and to analyze online chatting, including on QQ and WeChat (weekend), as well as food intake based on age. Data processing and statistical analyses were performed using SAS 9.2 software (SAS Institute, Cary, NC, USA). All tests were two-sided, and the level of significance was set at *p* < 0.05.

## 3. Results

### 3.1. Study Population

A total of 225 children and adolescents (aged 6–17 years) were included in the study, including 127 (56.4%) males and 98 (43.6%) females. The prevalence of obesity was 10.5% among children and adolescents living in urban areas, which was higher than those living in rural areas (5.9%) (Z = 10.192; *p* = 0.001). The prevalence of overweightness and obesity was 17.3% among male children and adolescents, which was higher than females (10.2%), but without significant difference (c^2^ = 4.861; *p* = 0.088).

### 3.2. Time of Sedentary Behavior and Food Intake 

Children and adolescents who watched TV on weekdays decreased their intake of vegetables (Spearman’s rho = −0.296; *p* < 0.05). Children and adolescents who watched movies or TV shows online or on a smartphone on weekends increased their intake of staple foods (Spearman’s rho = −0.486; *p* < 0.05), mainly instant noodles, fried pasta, and sweet potatoes (Spearman’s rho = 0.468, 0.575, and 0.513; *p* < 0.05). Children and adolescents who browsed online on weekdays increased their intake of milk and snacks (Spearman’s rho = 0.528 and 0.503; *p* < 0.05), mainly whole-fat liquid milk and fried potato chips (French fries or other fried snacks) (Spearman’s rho = 0.455 and 0.568; *p* < 0.05). Children and adolescents who chatted online, including on QQ and WeChat, on weekdays and on weekends increased their intake of vegetables (Spearman’s rho = 0.409 and 0.530; *p* < 0.05). Children and adolescents who chatted online, including on QQ and WeChat, on weekends increased their intake of staple foods, beans, and snacks (Spearman’s rho = 0.310, 0.415, and 0.427; *p* < 0.05), mainly rice, instant noodles, fried pasta, sweet potatoes, soybean milk tofu skin, fried potato chips (French fries or other fried snacks), nuts (peanuts, melon seeds, pumpkin seeds, watermelon seeds, and other melon seeds), and sweet cookies (buns, cakes, and moon cakes) (Spearman’s rho = 0.354, 0.465, 0.323, 0.313; 0.278, 0.461, 0.27, 0.551, and 0.296; *p* < 0.05). Children and adolescents who played computer or smartphone games on weekdays increased their intake of vegetables (Spearman’s rho = 0.545; *p* < 0.05), and who played computer or smartphone games on weekends, increased their intake of beans and snacks (Spearman’s rho = 0.421 and 0.450; *p* < 0.05); the snacks consumed were mainly fried potato chips (Spearman’s rho = 0.412; *p* < 0.05). Children and adolescents who did homework on weekends increased their intake of fruits, snacks, and water and beverages (Spearman’s rho = 0.227, 0.359, and 0.320; *p* < 0.05); the snacks consumed were mainly fried potato chips and sweet cookies (buns, cakes, and moon cakes) (Spearman’s rho = 0.299 and 0.206; *p* < 0.05), and the beverages were mainly water, cola, and 100% juice (Spearman’s rho = 0.31, 0.271, and 0.291; *p* < 0.05). Children and adolescents who read (books, newspapers, and magazines), wrote, or drew on weekdays increased their intake of eggs (Spearman’s rho = 0.358; *p* < 0.05), primarily fresh eggs (eggs, duck eggs, goose eggs, and quail eggs). Children and adolescents who read (books, newspapers, and magazines), wrote, or drew on weekends increased their intake of beans, eggs, and snacks (Spearman’s rho = 0.297, 0.257, and 0.253; *p* < 0.05); the beans consumed were mainly dried soybeans (soybeans, green beans, or black beans), and snacks were mainly nuts (peanuts, melon seeds, pumpkin seeds, watermelon seeds, and other melon seeds) (Spearman’s rho = 0.282; *p* < 0.05) (Table 1).

Linear regression showed that children and adolescents who chatted online (on weekends), including on QQ and WeChat, increased their intake of beans, vegetables, and snacks (t = 2.804, −2.516, and −2.641; *p* < 0.05) (Table 2).

### 3.3. Time Taken to Go to School by Bus or Subway and Food Intake

Children and adolescents who went to school by bus or subway increased their intake of staple foods (Spearman’s rho = 0.453; *p* < 0.05), mainly rice, instant noodles, and sweet potatoes (Spearman’s rho = 0.394, 0.536, and 0.630; *p* < 0.05). Children and adolescents who went to school by bus or subway increased their intake of beans (Spearman’s rho = 0.444; *p* < 0.05), mainly via soybean milk and tofu skins (Spearman’s rho = 0.408 and 0.485; *p* < 0.05). Children and adolescents who went to school by bus or subway increased their intake of vegetables (Spearman’s rho = 0.444; *p* < 0.05). Children and adolescents who went to school by bus or subway increased their intake of meat (Spearman’s rho = 0.479; *p* < 0.05), mainly processed meat products (sausage, ham sausage, or lunch meat) and fish and shrimp (Spearman’s rho = 0.408 and 0.485; *p* < 0.05). Children and adolescents who went to school by bus or subway increased their intake of snacks (Spearman’s rho = 0.479; *p* < 0.05), mainly nuts and sweet cookies (buns, cakes, and moon cakes) (Spearman’s rho = 0.541 and 0.366; *p* < 0.05). Children and adolescents who went to school by car, taxi, or motorcycle increased their intake of staple foods (Spearman’s rho = 0.294; *p* < 0.05), mainly fried pasta and sweet potatoes (Spearman’s rho = 0.329 and 0.321; *p* < 0.05). Children and adolescents who went to school by car, taxi, or motorcycles increased their intake of vegetables (Spearman’s rho = 0.354; *p* < 0.05) (Table 3).

Linear regression shows that children and adolescents who went to school by bus or subway increased their intake of snacks (t = −3.068; *p* < 0.05) (Table 4).

## 4. Discussion

To our knowledge, this is the first study to explore the relationship between sedentary behavior and food intake among children and adolescents under current socioeconomic and demographic circumstances in Zhejiang Province, China. We demonstrated the relationship between the duration of sedentary behavior and food intake, and the relationship between the time taken to go to school by bus or subway and food intake. 

The main finding of this study is that sedentary behavior affects the types of food consumed in children and adolescents’ daily diets. This finding may partly explain why higher amounts of time in sedentary behavior are strongly associated with overweightness and obesity, as reported in previous studies [1,4]. The prevalence of overweightness and obesity in children and young people has increased significantly worldwide. Over 340 million children and adolescents (aged 5–9 years) were shown to be overweight or obese worldwide, respectively [8]. The primary causes of overweightness and obesity in children can be traced to various lifestyle behaviors related to an imbalance between calorie intake and energy expenditure [9]. Health behaviors vary significantly based on socioeconomic and demographic circumstances [10]. There are strong adverse associations between time spent in sedentary behaviors and different health indicators, including the increased likelihood of being overweight or obese [10]. Sedentary behaviors have independent effects on total energy expenditure, weight, and metabolic variables [11]. The rapid evolution of socioeconomic has influenced children’s and adolescents’ sedentary behavior [12]. This evolution, nevertheless, varied from country to country. Internet and computer use are increasingly common sedentary behaviors during leisure time, which have the potential to impact negatively on health outcomes [13]. However, little is known about the extent to which food intake is associated with time spent in sedentary behavior. Exploring the relationship between sedentary behavior and food intake among children and adolescents instead of the isolated effects of food intake will help develop interventions to further target lifestyle behaviors simultaneously. A study on sedentary behavior, physical activity, and energy-dense food intake in six-year-old children showed that children from highly educated mothers or high-income households were more likely to be allocated to the “relatively healthy lifestyle” cluster, while children with low-education mothers or from low-income households were more likely to be allocated in the “high screen time and physically inactive” cluster [14]. Another study carried out in the WHO European region found that low-socioeconomic-status (SES) children were less likely to participate in sports clubs and more likely to have more than 2 h/day of screen time [15].

Sedentary behavior is associated with poorer diet quality among children. Reduced diet quality includes the more frequent consumption of high amounts of sugar, fat, and salt, as well as foods with low nutrient density. Physical activity has been proposed to be a stress-induced eating repressor, which may eventually limit junk food consumption [16]. Screen time exposure could act as a mediator in the association between physical activity and junk food consumption. Screen time exposure may play an important role, not only because it may be associated with poorer dietary patterns, but also because it may act as a displacer of healthier habits, such as exercising [17]. A Norwegian study tracked physical activity from 2005/2006 to 2011/2012 using accelerometers and found that both children and adolescents replaced time spent in light physical activity, with time spent being sedentary [18]. A study in Korea found that prolonged smartphone use was associated with a higher prevalence of the frequent intake of instant noodles, and proposed that both the duration and content type of smartphone use are independently associated with dietary risk factors among adolescents [19]. Prolonged internet use during leisure time was associated with the high intake of instant noodles and chips/crackers [20]. Similarly, a Spanish study reported that children spending at least one hour on daily leisure screen time had a higher prevalence of high-frequency sweet and snack intake than children exposed for less than one hour [21]. A positive association between TV viewing and the consumption of pizza, fried foods, sweets, and snacks was also demonstrated [22]. Unhealthy behavior appeared to aggregate in this group of Saudi adolescents, as healthful dietary habits (the higher consumption of breakfast, fruit, vegetables, and milk/dairy) were mostly associated with physical activity, whereas unhealthful dietary habits (the higher consumption of sugar-sweetened drinks, fast food, cake/donuts, and energy drinks) were related most to screen time [23]. In this study, we found that children and adolescents who watched movies or TV programs online or on their smartphones on weekends, as well as those who chatted online on weekends, including on QQ and WeChat, increased their intake of instant noodles and fried pasta; furthermore, those who browsed and chatted online on weekends, including on QQ and WeChat, as well as those who played computer or smartphone games, increased their intake of fried potato chips (French fries or other fried snacks). The intake of unhealthy junk food has increased in recent years, possibly because it is so easy to eat [22]. In this study, children and adolescents who chatted online on weekends, including on QQ and WeChat, increased their intake of tofu skins, mostly spicy strips; these foods are high in oil and salt, and have a low nutrient density. Although there must be other confounders that have not been considered in this study, we conducted linear regression to analyze online chatting and food intake based on age. We found that children and adolescents who chatted online (on weekends), including on QQ and WeChat, increased their intake of beans, vegetables, and snacks.

Contrary to what might be expected, we found that children and adolescents who browsed online on weekdays increased their intake of whole-fat liquid milk. While it is difficult to explain this outcome, it could be possibly due to a higher socio-economic profile. With the penetration of the Internet increasing, its users become more representative of the general population, and socio-economic differences are diminishing [24]. A study found that socio-demographic profiles emerged when Internet and computer use were categorized into different levels of usage. It was indicated that participants with low Internet and computer use during leisure time had the highest socio-economic profiles, were engaged in other sedentary behaviors for less time, and were slightly more likely to take part in more leisure time physical activity. On the other hand, participants with high Internet and computer use during leisure time had lower socio-economic profiles, were engaged in other sedentary behaviors for more time, and had a higher BMI. This suggests that different levels of Internet and computer use during leisure time are related to different socio-demographic profiles and health behaviors [25]. In this study, children and adolescents who browsed online on weekdays may be influenced by their own learning requirements and their parents’ daily learning requirements. At this point, parents may also be more focused on their children’s diet than on weekends. In response to this finding, we advocate that beverages should not be stored at home and that milk should be prepared to facilitate children’s choice of milk as a supplement to their three-meal diet during resting time. At the same time, yogurt, fruits, and other snacks can be used at home to improve the quality of children’s meals during sedentary time.

Children and adolescents who walked to go school had better nutritional habits vs. children and adolescents who went to school using motorized transports (who had poor nutritional habits). Adolescents who were using public/private modes of transportation to travel to school showed a significant association with overweightness/obesity [26]. Students living in neighborhoods of low walk frequency often fail to meet physical activity recommendations [27], and poor neighborhood social cohesion may affect nutritional behavior through physical activity [28]. Transportation methods can affect various types of food consumed. The linear regression conducted in our study showed that children and adolescents who went to school by bus or subway increased their intake of snacks, although there must be other confounders that have not been considered in this study. A study found that adolescents’ duration of exposure to unhealthy food outlets between home and school had a significant effect on the likelihood of junk food being purchased [29]. In this study, we found that, in terms of the mode of transportation for going to school and going home, children and adolescents taking buses and subways increased their intake of rice, instant noodles, sweet potatoes, soybean milk, tofu skin, processed meat products (sausage, ham sausage, or lunch meat), fish, shrimp and vegetables, and nuts and sweet cookies (buns, cakes, dim sum, and moon cakes). Eating ‘on the go’ is associated with less healthy food choices for adolescents and young adults [30]. Programs to encourage transport to schools should also consider provide information on eating environments.

Further prospective studies should be carried out to confirm our results in order for governments to consider implementing comprehensive policies directed not only to reduce sedentary time, but also to promote healthier habits overall, which may impact children and adolescents’ future health. Specific programs may include school-based interventions to encourage children to change their lifestyles or information campaigns for parents to inform them on the consequences of the potential harmful effects of sedentary behavior, and family food environments should include a fruit bowl or vegetable platter, full of attractive and varied fruits and vegetables, placed near computers or smartphones.

### Strengths and Limitations

All data are cross-sectional, and a causal effect of sedentary behavior on the frequency of kinds of food consumption cannot be established and reverse causality should not be discarded. Secondly, evidence shows that children from low-income families are more likely to spend longer periods using screens and follow worse dietary patterns [31]. In this sense, parents with longer working hours may offer their children more readily available options that require less of their time, such as mobile devices to entertain them and junk food to feed them. This study has not yet investigated the working hours of parents, which may be an influencing factor. Future research should conduct multi-factor regression to increase the strength of the data analysis. To further our understanding of this complex relationship between sedentary behavior time and diet quality, future research should include interventions which provide information about the possible underlying factors. All dietary intake methodologies, such as the use of food frequency questionnaires or dietary recall, have their limitations, which may lead to either incomplete or inaccurate reporting, although limitations with regards to the accuracy of dietary intake data may still be present, even in high-quality studies.

## 5. Conclusions

Sedentary behavior affects the types of food consumed in the daily diets of children and adolescents. The children and adolescents who browse online on weekdays increased their intake of whole-fat liquid milk, but also increased their intake of foods with high fat, high salt, and low nutrient density. The children and adolescents who take buses and subways increased their intake of low-nutrition quality products. Public awareness efforts should focus on reducing the consumption of low-nutrition quality products and nutritional education. Preventive strategies can use this information to develop snack guidelines for sedentary behavior, such as increasing the intake of milk and reducing the intake of low-nutrient-density foods.

## Figures and Tables

**Table 1:** Sedentary behaviors and daily food intake.

Sedentary Behavior (h/Day)	Food Category (Spearman’s Rho)								
	Staple Food	Beans	Vegetables	Fruits	Dairy	Meat	Eggs	Snacks	Water and Beverages
Watching TV (mid-week)	0.018	0.143	−0.296 *	0.255	0.107	0.24	0.121	0.232	0.251
Watching TV (on weekends)	0.183	0.193	0.008	0.008	−0.097	−0.006	−0.132	0.25	0.052
Watching movies or TV shows online or on a smartphone (mid-week)	0.303	−0.05	0.232	−0.368	0.04	0.474	0.25	0.442	−0.013
Watching movies or TV shows online or on a smartphone (on weekends)	0.486 *	0.26	0.226	−0.121	−0.157	0.196	−0.192	0.39	−0.168
Online browsing (mid-week)	0.020	0.2	0.038	0.083	0.528 *	0.356	0.164	0.503 *	−0.111
Online browsing (on weekends)	0.261	0.296	0.245	0.071	0.152	0.219	0.036	0.411	−0.113
Online chat, including QQ and WeChat (mid-week)	−0.014	0.184	0.409 *	−0.251	−0.117	−0.15	0.163	0.259	0.03
Online chat (on weekends), including QQ and WeChat	0.310 *	0.415 *	0.53 *	−0.101	−0.052	0.152	−0.141	0.427 *	0.028
Playing computer or smartphone games (mid-week)	0.046	0.229	0.545 *	0.064	−0.277	−0.144	0.058	0.220	−0.243
Playing computer or smartphone games (on weekends)	0.057	0.421 *	0.345	0.168	−0.113	0.058	−0.037	0.450 *	−0.042
Completing homework (mid-week)	0.164	0.177	−0.103	0.227 *	0.066	0.104	0.149	0.359 *	0.320 *
Completing homework (on weekends)	0.790	0.286	0.314	0.031	0.143	0.204	0.791	0.245	0.243
Reading (books, newspapers, and magazines), writing, or drawing (mid-week)	−0.069	0.198	0.094	0.049	−0.062	0.120	0.358 *	0.231	0.208
Reading (books, newspapers, and magazines), writing, or drawing (on weekends)	−0.050	0.297 *	0.182	0.000	−0.014	0.049	0.257 *	0.253 *	0.278

* *p* < 0.05.

**Table 2:** Linear regression of online chatting (on weekends), including on QQ and WeChat, and food intake.

	Unstandardized Coefficients		Standardized Coefficients			95% CI for B	
Factors	B	Std.E	β	t	*p*	Lower Bound	Upper Bound
Staple food	−0.011	0.016	−0.090	−0.714	0.478	−0.043	0.021
Beans	0.067	0.024	0.382	2.804	0.007	0.019	0.116
Vegetables	−0.025	0.010	−0.353	−2.516	0.015	−0.045	−0.005
Snacks	−0.057	0.022	−0.357	−2.641	0.011	0.101	−0.014
Age	−3.710	1.178	−0.364	−3.149	0.003	−6.076	−1.345

**Table 3:** Time taken to go to school by bus or subway and daily food intake.

Sedentary Behavior (h/Day)	Food Category (Spearman’s Rho)								
	Staple Food	Beans	Vegetables	Fruits	Dairy	Meat	Eggs	Snacks	Water and Beverages
Walk	0.076	0.128	0.2	0.204	0.178	0.3	0.276	0.3	0.313
Bus and subway	0.453 *	0.444 *	0.444 *	−0.087	−0.087	0.479 *	−0.126	0.479 *	−0.096
Cars, taxis, and motorcycles (electric vehicles)	0.294 *	0.201	0.354 *	0.109	0.207	0.125	0.15	0.278	0.03

* *p* < 0.05.

**Table 4:** Linear regression of time taken to go to school by bus or subway and daily food intake.

	Unstandardized Coefficients		Standardized Coefficients			95%CI for B	
Factors	B	Std.E	β	t	*p*	Lower Bound	Upper Bound
Staple Food	0.005	0.022	0.032	0.229	0.821	−0.040	0.049
Beans	−0.090	0.050	−0.348	−1.790	0.087	−0.194	0.014
Vegetables	−0.029	0.015	−0.311	−1.952	0.063	−0.060	0.002
Meat	0.019	0.031	0.098	0.623	0.540	−0.045	0.084
Snacks	−0.186	0.061	−0.456	−3.068	0.005	−0.312	−0.061
Age	0.329	1.420	0.030	0.232	0.819	−2.609	3.266

## Data Availability

The datasets analyzed in this study are available from the corresponding authors on reasonable request.
